# Increased Bioplastic Production with an RNA Polymerase Sigma Factor SigE during Nitrogen Starvation in *Synechocystis* sp. PCC 6803

**DOI:** 10.1093/dnares/dst028

**Published:** 2013-07-15

**Authors:** Takashi Osanai, Keiji Numata, Akira Oikawa, Ayuko Kuwahara, Hiroko Iijima, Yoshiharu Doi, Kan Tanaka, Kazuki Saito, Masami Yokota Hirai

**Affiliations:** 1 RIKEN Center for Sustainable Resource Science, 1-7-22 Suehiro-cho, Tsurumi-ku, Yokohama, Kanagawa 230-0045, Japan; 2 PRESTO, Japan Science and Technology Agency (JST), 4-1-8 Honcho, Kawaguchi, Saitama 332-0012, Japan; 3 RIKEN Biomass Engineering Program, 2-1 Hirosawa, Wako, Saitama351-0198, Japan; 4 Chemical Resources Laboratory, Tokyo Institute of Technology, 4259, Nagatsuta-cho, Midori-ku, Yokohama, Kanagawa 226-8503, Japan; 5 Graduate School of Pharmaceutical Sciences, Chiba University, 1-8-1 Inohana, Chuo-ku, Chiba 260-8522, Japan

**Keywords:** cyanobacteria, gene expression, metabolomics, nitrogen starvation, sugar metabolism

## Abstract

Because cyanobacteria directly harvest CO_2_ and light energy, their carbon metabolism is important for both basic and applied sciences. Here, we show that overexpression of the sigma factor *sigE* in *Synechocystis* sp. PCC 6803 widely changes sugar catabolism and increases production of the biodegradable polyester polyhydroxybutyrate (PHB) during nitrogen starvation. *sigE* overexpression elevates the levels of proteins implicated in glycogen catabolism, the oxidative pentose phosphate pathway, and polyhydroxyalkanoate biosynthesis. PHB accumulation is enhanced by *sigE* overexpression under nitrogen-limited conditions, yet the molecular weights of PHBs synthesized by the parental glucose-tolerant and *sigE* overexpression strain are similar. Although gene expression induced by nitrogen starvation is changed and other metabolites (such as GDP-mannose and citrate) accumulate under *sigE* overexpression, genetic engineering of this sigma factor altered the metabolic pathway from glycogen to PHB during nitrogen starvation.

## 1. Introduction

Cyanobacteria can utilize light energy via two photosystems. Carbon dioxide is fixed via the Calvin-Benson cycle, producing sugar phosphate, followed by production of metabolites including glycogen as a carbon and energy sources.^1,2^ Stored glycogen is oxidized by glycogen catabolic enzymes such as glycogen phosphorylases (encoded by *glgP*) or isoamylases (encoded by *glgX*) (Supplementary Fig. S1).^3^ Under heterotrophic conditions, the glucose (or glucose-phosphate) are degraded mainly through the oxidative pentose phosphate (OPP) pathway.^4^ Under mixotrophic conditions, glucose is degraded via glycolysis and CO_2_ is concomitantly fixed via the Calvin cycle, providing organic acids such as pyruvate.^4^ The pyruvate is used to biosynthesize organic acids (including amino acids), to generate reducing power, or to assist nitrogen assimilation through the tricarboxylic acid cycle. From previous studies, it is known that many sugar catabolic genes are activated by the sigma factor SigE in *Synechocystis* 6803.^5^ *Synechocystis* genome contains nine sigma factors (SigA–I), and SigE belongs to Group 2 sigma factor that consists of four sigma factors (SigB–E).^5^ Overexpression of *sigE* enhances metabolite levels of acetyl-CoA and organic acids such as citrate,^6^ implying utilization of this sigma factor for metabolic engineering. The expression of *sigE* is increased by nitrogen depletion;^7,8^ however, the implication of SigE into metabolite profiling during nitrogen starvation have not been described.

Polyhydroxyalkanoates (PHAs) are a class of polyesters, stored by many bacteria as carbon and energy sources. PHAs have attracted industrial interest, because they are naturally biodegradable.^9^ PHAs, consisting of short-chain-length or medium-chain-length monomers, are the constituents of >150 identified hydroxyalkanoates.^10,11^ Several PHAs, such as poly[(*R*)-3-hydroxybutyrate] (PHB, a homopolymer of 3-hydroxybutyrate) and poly[(*R*)-3-hydroxybutyrate-*co*-(*R*)-3-hydroxyhexanoate] (a co-polymer of 3-hydroxybutyrate and 3-hydroxyhexanoate), are industrially produced today (for example, KANEKA Biopolymer AONILEX). PHA granules are subcellular complex, consisting of a polyester core, associated proteins such as PHA synthases, phasins, PHA depolymerases, and regulatory proteins.^11–14^ Life cycle assessment studies have suggested that using PHB in place of conventional petrochemical polymers (such as film products and disposable items) lowers environmental impacts.^15^ PHAs are potentially applicable to medical research such as surgical sutures, vein valves, and targeted drug delivery,^12^ though their expensive production remains a major obstacle to their universal development.

PHAs, particularly PHB, accumulate in several cyanobacterial strains including *Synechocystis* sp. PCC 6803 (we herein designate this strain *Synechocystis* 6803).^16^ Levels of PHB in cyanobacteria increase under nitrogen or phosphorus starvation.^17^ Addition of external carbon sources such as acetate enhances PHB accumulation in *Synechocystis* 6803 and other cyanobacteria.^18–20^ The biosynthetic pathway of PHB in *Synechocystis* 6803 is currently well understood. Acetoacetyl-CoA is formed from two molecules of acetyl-CoA by the enzyme β-ketothiolase (encoded by *phaA*). Then, d-3-hydroxybutyryl-CoA is synthesized, catalysed by acetoacetyl-CoA reductase (encoded by *phaB*).^20^ d-3-hydroxybutyryl-CoA is also polymerized to PHB by a PHA synthase, comprising PhaC and PhaE.^16^ PHA synthase activity in cyanobacteria is up-regulated in the presence of acetyl phosphate.^18^ Reverse genetic analysis has revealed that a protein designated Sll0783, which is conserved among PHB-producing bacteria and which is expressed under nitrogen depletion stress, is essential for retaining PHA synthase activity during prolonged nitrogen starvation.^21^ Disruption of sll0461, encoding gamma-glutamyl phosphate reductase, or sll0565, encoding a hypothetical protein, increases PHB accumulation in standard growth media.^22^ Transcription levels of *phaA*, *B*, *C*, and *E* have been shown to increase following nitrogen depletion.^8^ Expression of all four genes peaks 6 h after nitrogen depletion, then gradually decreases, but remains above the nitrogen-replete levels for a minimum of 120 h.^21^ To date, transcriptional regulator(s) for controlling PHA biosynthetic gene expression have not been found in cyanobacteria. It is noteworthy that introducing external *pha* regulons into *Synechocystis* 6803 increases enzymatic activities of PHA synthases, but does not enhance PHB accumulation.^19^

Here, we show that genetic engineering of *sigE* resulted in large change of sugar metabolism and increased PHB accumulation during nitrogen starvation, indicating that sigma factor is useful for metabolic engineering of cyanobacteria.

## 2. Materials and methods

### 2.1. Bacterial strains and culture conditions

The glucose-tolerant (GT) strain of *Synechocystis* sp. PCC 6803 (isolated by Williams)^23^ and the *sigE*-overexpressing strain GOX50 were grown in modified BG-11 medium; that is, BG-11_0_ liquid medium containing 5 mM NH_4_Cl (buffered with 20 mM 4-(2-hydroxyethyl)-1-piperazineethanesulfonic acid - potassium hydroxide-KOH, pH 7.8). Liquid cultures were bubbled with 1% (v/v) CO_2_ in air, at 30°C under continuous white light (around 50–70μmol photons m^–2^ s^–1^).^24^ The *sigE*-overexpressing strain was generated in a GT background, as described previously.^6^ Growth and cell densities were measured at *A*_730_ with a Hitachi U-3310 spectrophotometer. In our experimental condition, *A*_730_ = 1.0 corresponds to 6.9 μg/ml chlorophyll *a*.

### 2.2. RNA isolation and quantitative real-time PCR

Pre-cultured cells were diluted to *A*_730_ = 0.2 in 70 ml BG-11_0_ medium supplemented with 5 mM NH_4_Cl. Cells were collected by filtration after 1 day of cultivation and re-suspended in BG-11_0_ medium, followed by cultivation for 2 or 4 h. RNA isolation and quantitative real-time PCR were performed, as previously described^25^ using primers listed in Supplementary Table S1. The expression level of *rnpB* (encoding RNaseP subunit B) was used as an internal standard as previously described.^21^

### 2.3. Purification of GST-tagged PhaA, B, C, and E

The regions of the *Synechocystis* 6803 genome encoding *phaA*, *B*, *C*, and *E* were amplified by PCR using relevant primers (Supplementary Table S1). The amplified DNA fragments were digested with *Eco*RI and *Sal*I, and inserted into the *Eco*RI–*Xho*I sites of pGEX5X-1 (GE Healthcare Japan, Tokyo, Japan) using the Mighty Mix ligation reagent kit (Takara Bio., Shiga, Japan). Purification was performed as described previously (Supplementary Fig. S2).^26^

### 2.4. Glycogen measurement

Pre-cultured cells were diluted to *A*_730_ = 0.2 in 70 ml BG-11_0_ medium supplemented with 5 mM NH_4_Cl. Cells were collected by filtration after 1 day of cultivation and re-suspended in BG-11_0_ medium, followed by cultivation for 1 or 3 days. Glycogen levels were quantified as described previously.^25^

### 2.5. Production of antisera and immunoblotting

We have previously produced antisera against glucose-6-phosphate dehydrogenase (G6PD), 6-phosphogluconate dehydrogenase (6PGD), GlgX(slr0237), GlgX(slr1857), and GlgP(sll1356).^6^ To make antisera against PHA biosynthetic enzymes, glutathione-S-transferase (GST)-PhaB and GST-PhaE were injected into rabbits, whereas GST-PhaC was injected into a rat [procedures were performed by Tampaku Seisei Kogyo Co. Ltd, (Gunma, Japan)]. Antisera against GlgP(slr1367) and PhaA were produced by injecting peptides NH_2_-EIIHNEKNQPK-COOH or NH_2_-CDGLTDSTNGEGMGEQ-COOH, respectively, into rabbits. Pre-cultured cells were diluted to *A*_730_ = 0.2 in 70 ml BG-11_0_ medium supplemented with 5 mM NH_4_Cl. Cells were collected by filtration after 1 day of cultivation and re-suspended in BG-11_0_ medium, followed by cultivation for 1 or 3 days. Immunoblotting was performed as described previously.^25^

### 2.6. Analysis of G6PD and 6PGD activities

Cells were similarly cultivated with immunoblotting. G6PD and 6PGD activities were measured as described previously.^6^ One unit of G6PD or 6PGD activity was defined as the formation of 1μmol NADPH per minute under the specified experimental conditions.

### 2.7. Extraction and purification of PHAs

Pre-cultured cells were diluted to *A*_730_ = 0.2 in 70 ml BG-11_0_ medium supplemented with 3 or 5 mM NH_4_Cl. For first experiment, cells grown with 3 mM NH_4_Cl were cultured for 9 days (nitrogen sources were naturally exhausted). For second experiment, cells grown with 5 mM NH_4_Cl were collected by filtration after 1 day of cultivation and re-suspended in BG-11_0_ medium, followed by cultivation for 3 days. Cells were collected by centrifugation at 18 000 × *g* for 2 min. Cells were freeze-dried with FDU-2200 (EYELA, Tokyo, Japan) at −80°C for 3 days. Freeze-dried cell weights were determined by weighing on an electronic balance. Cells from a single sample from each of the 140 or 280 ml cultures were used for PHA extraction for the first or second experiment, respectively. Each 100–150 mg freeze-dried cells were suspended in 7 ml chloroform in a 12-ml glass tube and disrupted by sonication for 80 s (VC-750 sonicator, EYELA). The suspension was incubated at 70°C for 4 days with additional sonication (four times during 4 days). The suspension was cooled to room temperature and filtrated with a mixed cellulose ester filter paper (Advantec, Tokyo, Japan). The filtrates were heated to 70°C and concentrated to 1 ml, followed by cooling to room temperature. Ten millilitres of hexane was added to the filtrates; the mixtures were then incubated overnight at room temperature. After centrifugation at 1000 × *g* for 30min, supernatants were discarded and the residual pellets were re-suspended in hexane, followed by centrifugation at 1000 × *g* for 30 min. Pellets were completely dried at 50°C and re-dissolved in 1 ml of chloroform at 70°C. Then, 10 ml of methanol was added and the suspensions were mixed by shaking and incubated overnight at room temperature. The PHA precipitates were collected in a 1.5-ml tube by centrifugation at 20 500 × *g* for 5 min, followed by drying in a vacuum evaporator at 65°C. The residual PHAs were weighed on an electronic balance as PHA amounts and preserved for gel permeation chromatography (GPC) and nuclear magnetic resonance (NMR) analysis.

### 2.8. Analysis of purified PHAs with GPC and NMR

GPC and NMR analyses were performed as described previously.^27^ The purified PHAs were subjected to GPC using the Shimadzu LC-10A GPC system (Shimadzu, Kyoto, Japan) with a Shodex K-806 column (Showa Denko, Tokyo, Japan) at 40°C. Chloroform was used as mobile phase at a flow rate of 0.8 ml/min. Polystyrene was used as the standard in molecular weight determination. Co-polymer compositions of purified PHAs were detected by NMR spectroscopy (JNM-Excalibur270; JEOL, Ltd, Tokyo, Japan). Each polymer was dissolved in 0.75 ml of CDCl_3_ and subjected to ^1^H NMR analysis. NMR spectra were recorded using an Excalibur270 spectrometer. Spectral inputs were 4.8-μs pulse width, 3.9-s pulse repetition, 5400-Hz spectra width, 16-K data points at 23°C, and 128 accumulations. Tetramethylsilane was used as an internal chemical shift standard.

### 2.9. Capillary electrophoresis–mass spectrometry analysis

Capillary electrophoresis–mass spectrometry analysis (CE–MS) was performed as previously described.^6^ The CE–MS system and conditions were as previously described.^28^

### 2.10. Statistical analysis

All statistical analyses were performed using the StatPlus:macLE software for MacOSX (Analyst Soft, Alexandria, VA, USA). *P*-values were determined using paired two-tailed *t*-tests. A 95% confidence interval was used to determine significance. The *P-*values are listed in Supplementary Table S2.

## 3. Results

### 3.1. Changes in levels of sugar catabolic enzymes during nitrogen starvation

**Figure 1.**
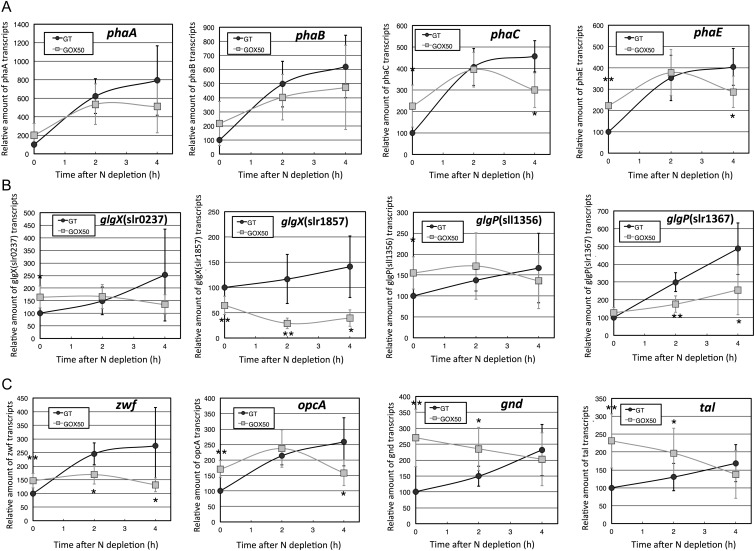
Quantitative real-time PCR analysis. Changing mRNA levels of genes involved in (A) PHA biosynthesis (left to right: *phaA*, *phaB*, *phaC*, and *phaE*), (B) glycogen catabolism [left to right: *glgX*(slr0237), *glgX*(slr1857), *glgP*(sll1356), and *glgP*(slr1367)], (C) the OPP pathway (left to right: *zwf*, *opcA*, *gnd*, and *tal*). Data represent mean ± SD from five to seven independent experiments. Levels were calibrated relative to that of the GT strain under nitrogen-replete conditions (set at 100%). Statistically significant differences between GT and GOX50 are marked by asterisks (Student's *t*-test; *P < 0.05, **P < 0.005).

Glycogen is degraded by isoamylase (*glgX*) and glycogen phosphorylase (*glgP*). *Synechocystis* genome contains two *glgX* genes (slr0237 and slr1857) and two *glgP* genes (sll1356 and slr1367).^6^ Previous transcriptome analysis revealed that the mRNA levels of genes for glycogen catabolism, the OPP pathway, and PHA biosynthesis increased during nitrogen starvation.^8^ The mRNA levels of two PHA biosynthetic genes (*phaC* and *phaE*), two glycogen catabolic genes (*glgX*(slr0237) and *glgP*(sll1356)), and four OPP pathway genes (*zwf*, *opcA*, *gnd*, and *tal*) in the *sigE* overexpression strain (designated GOX50) exceeded those of the parental wild type (GT) under nitrogen-replete conditions (Fig. 1). In the GT strain, the expression of all 12 genes increased under nitrogen depletion within 4 h (Fig. 1). In the *sigE* overexpression strain, most of these genes were less increased by nitrogen depletion, compared with the GT strain (Fig. 1). The mRNA levels of *phaA*, *phaB*, and *glgP*(slr1367) alone were elevated (relative to nitrogen-replete conditions) after 4 h of nitrogen depletion (Fig. 1). The induction of nitrogen-related genes such as *glnB* (encoding a nitrogen sensor PII protein) and *amt1* (encoding an ammonium transporter) were also less induced by *sigE* overexpression (Supplementary Fig. S3).

**Figure 2.**
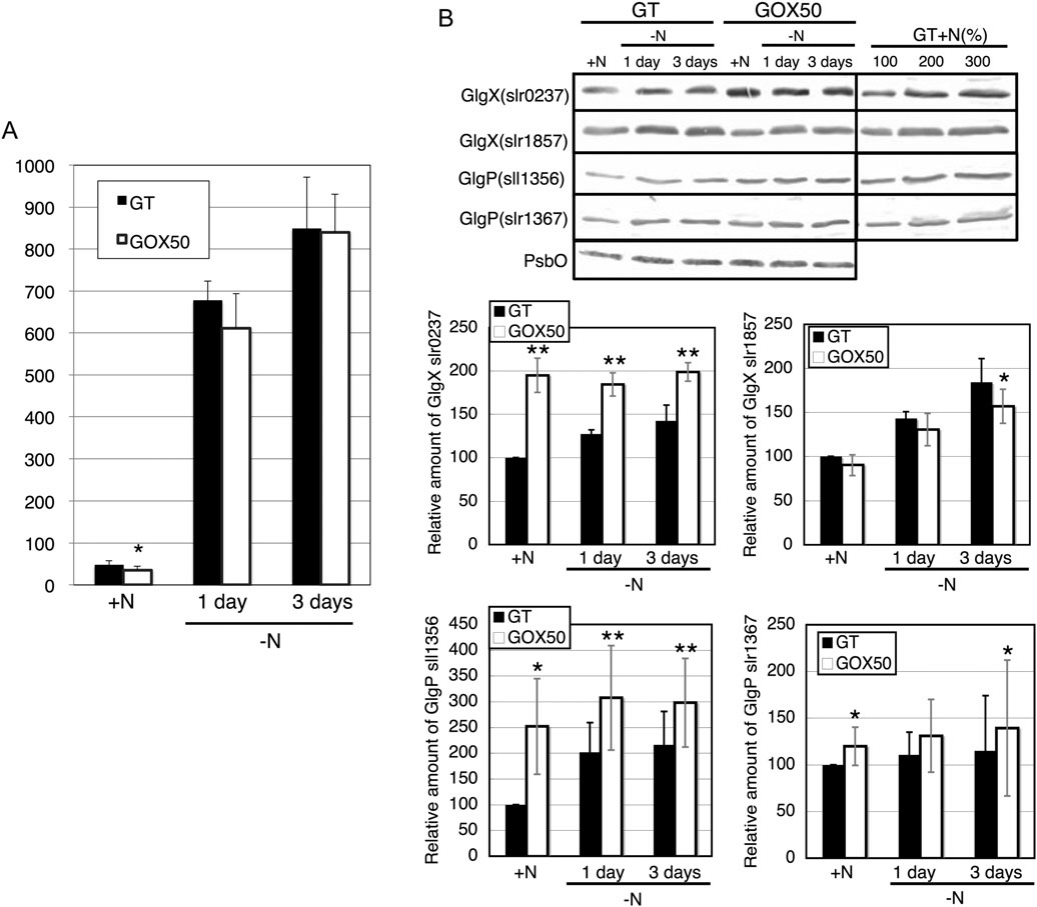
Levels of glycogen and glycogen catabolic proteins. (A) Levels of glycogen in the GT and GOX50 strains. Data represents mean ± SD results from five independent experiments. Glycogen levels were calibrated relative to that of the GT strain under nitrogen-replete conditions (set at 100%). Statistically significant differences between GT and GOX50 are marked by asterisks (Student's *t-*test; *P < 0.05). (B) Levels of GlgX (Slr0237 and Slr1857) and GlgP (Sll1356 and Slr1367) in the GT and GOX50 strains (total protein 14 μg). Bands show the relative expression of the four proteins for the two strains under nitrogen-replete conditions (+N) and after 1 and 3 days of nitrogen depletion (−N). Data represent mean ± SD results from five to seven independent experiments. Protein levels were calibrated relative to that of the GT strain under nitrogen-replete conditions (set at 100%).

We measured the glycogen amounts in the GT and *sigE* overexpression strain during nitrogen starvation. Consistent with the previous study,^6^ glycogen amount was reduced by *sigE* overexpression under nitrogen-replete conditions (Fig. 2A). During nitrogen starvation, however, glycogen similarly increased in the two strains (Fig. 2A). Levels of three glycogen catabolic enzymes, such as GlgX(slr0237), GlgX(slr1857), and GlgP(sll1356), increased under nitrogen depletion in the GT strain (Fig. 2B). Overexpression of *sigE* enhanced the levels of GlgX(slr0237), GlgP(sll1356), and GlgP(slr1367) under nitrogen-replete conditions (Fig. 2B). During prolonged nitrogen starvation, levels of GlgX(slr0237), GlgP(sll1356), and GlgP(slr1367) in the *sigE* overexpression strain remained higher than in the GT strain, although levels of GlgX(slr1857) were diminished (Fig. 2B).

**Figure 3.**
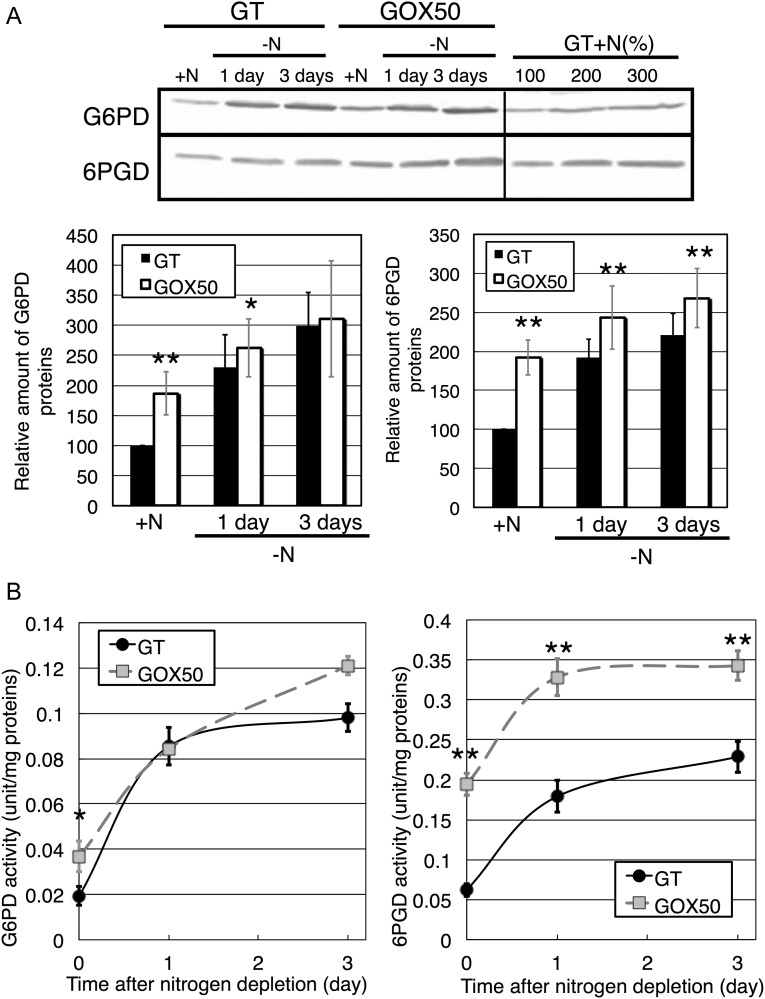
(A) Levels of G6PD and 6PGD in the GT and GOX50 strains under nitrogen-limited conditions, assayed by immunoblotting (total protein 12 μg). Bands show the relative expression of the two proteins under nitrogen-replete conditions (+N) and after 1 and 3 days of nitrogen depletion (−N). Data represent mean ± SD results from six independent experiments. Protein levels were calibrated relative to that of the GT strain under nitrogen-replete conditions (set at 100%). Statistically significant differences between GT and GOX50 are marked by asterisks (Student's *t-*test; *P < 0.05, **P < 0.005). (B) Enzyme activities of G6PD and 6PGD under nitrogen-replete and -depleted conditions. Data represent mean ± SD of values from three independent experiments.

Levels of G6PD and 6PGD, two key enzymes in the OPP pathway, increased under *sigE* overexpression in nitrogen-replete conditions (Fig. 3A). 6PGD protein levels remained higher in the *sigE* overexpression strain than in the GT during nitrogen starvation, whereas those of G6PD were similar in both strains after 3 days of nitrogen depletion (Fig. 3A). The enzymatic activities of both G6PD and 6PGD also increased under *sigE* overexpression in nitrogen-replete conditions (Fig. 3B). The enzymatic activities of 6PGD in the *sigE* overexpression strain remained higher for at least 3 days of nitrogen depletion, although the G6PD activities were similar between the two strains under these conditions (Fig. 3B).

### 3.2. Increased PHA biosynthetic enzymes under sigE overexpression

**Figure 4.**
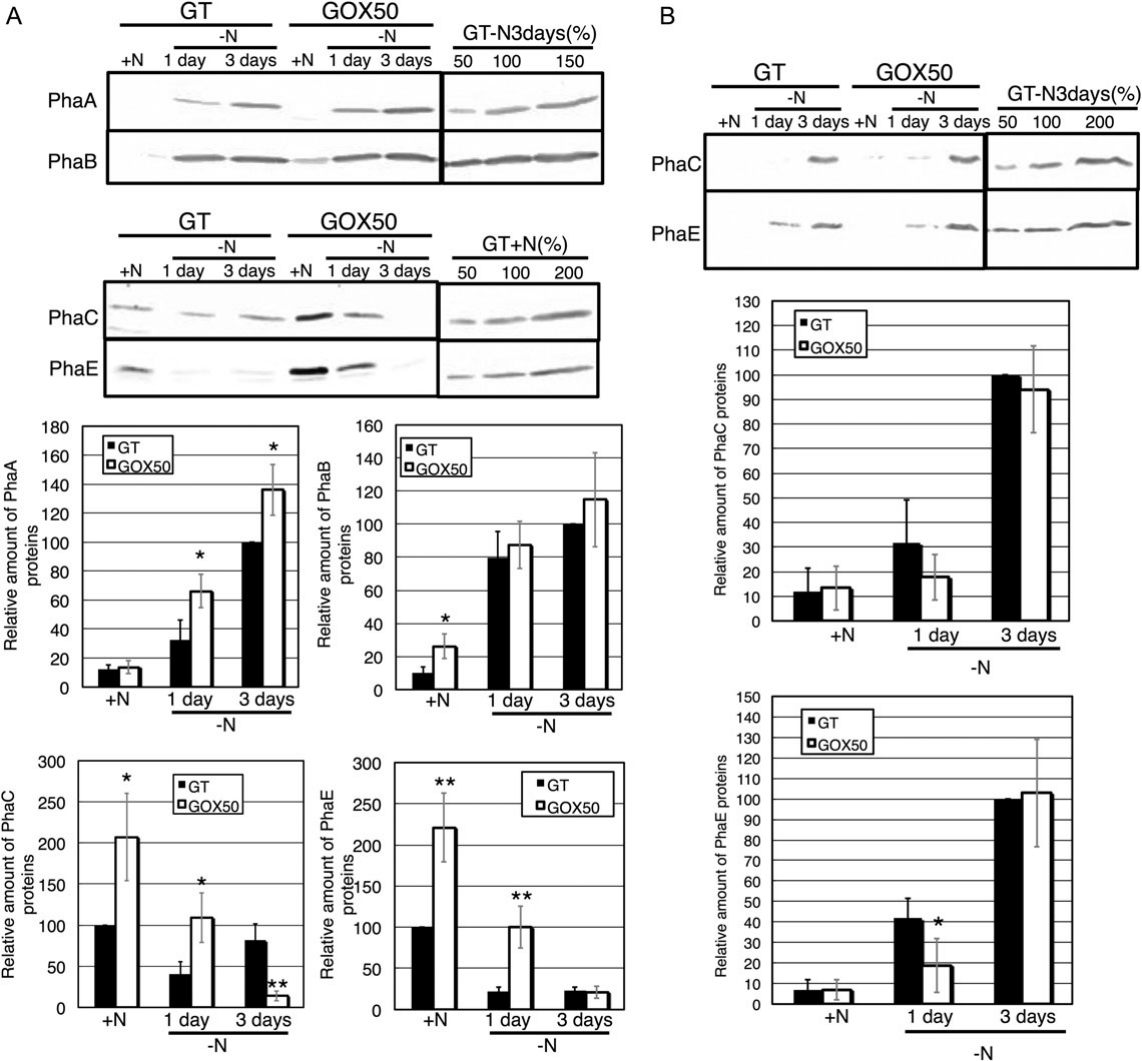
Levels of PHA biosynthetic enzymes. (A) Levels of PhaA, B, C, and E in the GT and GOX50 strains (total protein 12 μg). Bands show the relative expression of the four proteins for the two strains under nitrogen-replete conditions (+N) and after 1 and 3 days of nitrogen depletion (−N). Data represent mean ± SD results from five independent experiments. PhaA and B levels were calibrated relative to those of the GT strain 3 days after nitrogen depletion (set at 100%), while PhaC and E levels were calibrated relative to those of the GT strain under nitrogen-replete conditions (similarly set at 100%). Statistically significant differences between GT and GOX50 are marked by asterisks (Student's *t-*test; *P < 0.05, **P < 0.005). (B) PhaC and E protein levels in insoluble GT and GOX50 cell fractions. Data represent mean ± SD results from five independent experiments. PhaC and E levels were calibrated relative to that of the GT strain after 3 days of nitrogen depletion (set at 100%).

In the GT strain, the protein levels of PhaA and PhaB increased under nitrogen depletion, whereas those of PhaC and PhaE decreased (Fig. 4A). *sigE* overexpression increased the protein levels of PhaB, C, and E under nitrogen-replete conditions (Fig. 4A). PhaA levels in the *sigE* overexpression strain remained higher than those in the GT after 3 days of nitrogen depletion, although PhaB levels were similar between the two strains (Fig. 4A). Levels of PhaC and PhaE in the *sigE* overexpression strain were similar to or decreased (in the case of PhaC) after 3 days of nitrogen depletion, compared with the GT strain (Fig. 4A).

In a previous study, levels of PHB and mRNA of *phaC* and *phaE* were enhanced for at least 3 days under nitrogen deprivation (relative to nitrogen-replete conditions),^21^ inconsistent with our immunoblotting results. Since PHA synthase (comprising subunits PhaC and PhaE) is integrated into the PHB granule in other bacteria, we also measured the proteins levels of PhaC and PhaE within the insoluble factions of the cell. Under nitrogen-replete conditions, PhaC and PhaE proteins were not detected for both GT and *sigE* overexpression strains within insoluble fractions (Fig. 4B). During prolonged nitrogen starvation, however, PhaC and PhaE were detected in the insoluble factions at elevated levels in both strains (Fig. 4B).

### 3.3. sigE overexpression during nitrogen starvation enhances PHB levels (revealed by PHA analysis)

**Figure 5.**
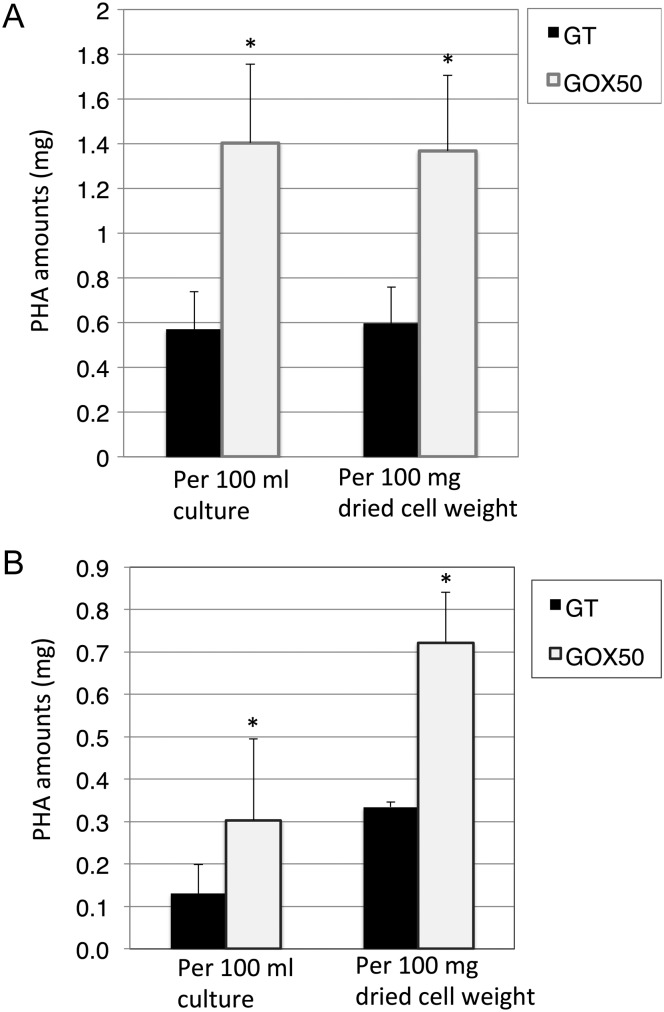
Quantification of purified PHAs from GT and GOX50 cells which (A) nitrogen sources were naturally exhausted and (B) transferred into nitrogen-free medium by filtration. Data represent mean ± SD from independent experiments (*n* = 3–6). Statistically significant differences between GT and GOX50 are marked by asterisks (Student's *t-*test; *P < 0.05). PHA weight per 100 ml culture (left) and per 100 mg dried cell weight (right).

Cells were cultured for 9 days in liquid medium supplemented with 3 mM NH_4_Cl. The colour of cell cultures turned from green to yellow during cultivation, indicating nitrogen sources were naturally exhausted (data not shown). The dried cell weight of the *sigE* overexpression strain exceeded that of the GT strain (data not shown). Within the same culture volume, PHA levels increased more in the *sigE* overexpression strain than in the GT strain (Fig. 5A). PHA contents per dried cell weight also increased by 2.3 times by *sigE* overexpression (Fig. 5A). PHAs were also extracted from the cells transferred into BG-11_0_ medium by filtration and cultivated for 3 days, whose condition is same as immunoblotting. PHA contents elevated by 2–3 times by *sigE* overexpression (Fig. 5B).

The purified PHAs (cultured in BG-11_0_ supplemented with 3 mM NH_4_Cl) were analysed by GPC and NMR. The number-average molecular weights and weight-average molecular weights (designated as *M_n_* and *M_w_*, respectively) of the PHAs were similar in the GT and *sigE* overexpression strains (*M_n_* was ∼135 kDa and *M_w_* was ∼190 kDa). The polydispersity indices of the purified PHAs from GT and the *sigE* overexpression strain were 1.4 ± 0.03 and 1.4 ± 0.08, respectively. Structures of the extracted PHAs were subsequently revealed by NMR as described previously.^29^ The NMR spectra of both strains were similar, demonstrating that, among the range of existing PHAs, both strains only produce PHB (Supplementary Fig. S4).

### 3.4. The metabolite levels related to sugar catabolism

**Figure 6.**
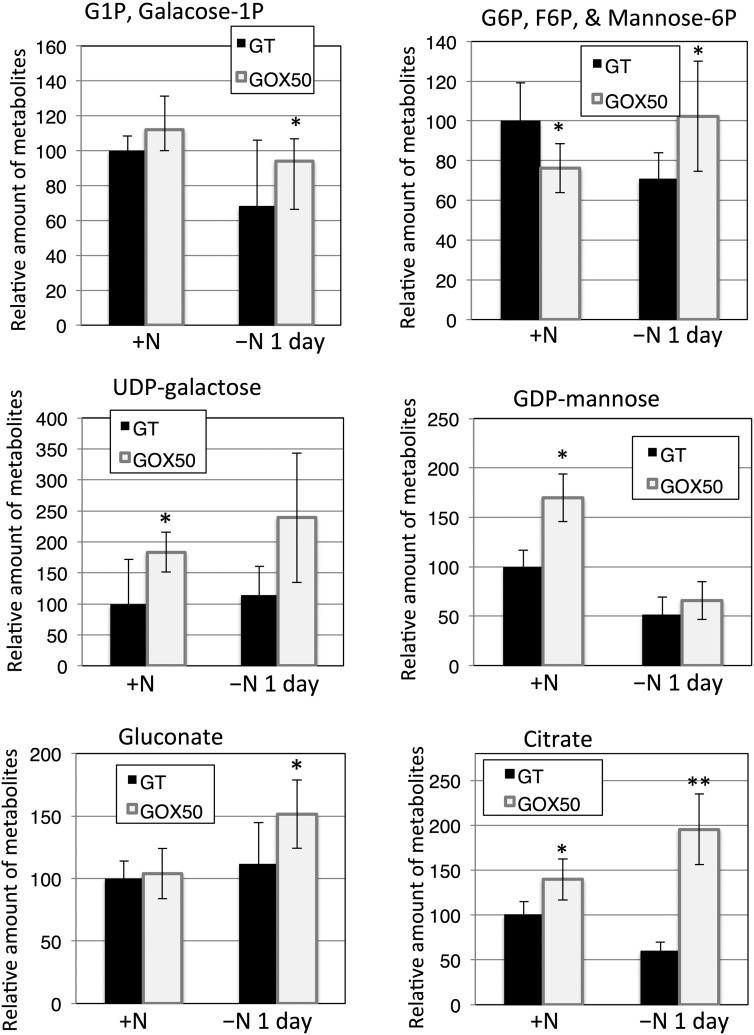
CE–MS analysis of a range of metabolites produced by GT and GOX50 cells. Data represent mean ± SD from five independent experiments. Levels were calibrated relative to that of the GT strain under nitrogen-replete conditions (set at 100%). Statistically significant differences between GT and GOX50 are marked by asterisks (Student's *t-*test; *P < 0.05, **P < 0.005).

CE–MS analysis was performed on cells grown in nitrogen-replete or -depleted conditions. Eighty-four peaks in the spectrum were attributable to known metabolites (Supplementary Table S3). Levels of sugar-phosphate metabolites, such as gluconate, glucose-1-phosphate/galactose-1-phosphate, and glucose-6-phosphate/fructose-6-phosphate/mannose-6-phosphate, in the *sigE* overexpression strain were higher than those in the GT strain after 1 day of nitrogen depletion (Fig. 6). Citrate levels increased in the *sigE* overexpression strain under both conditions (Fig. 6). *sigE* overexpression in nitrogen-replete media enhanced the production of nucleotide sugars, such as UDP-galactose and GDP-mannose (Fig. 6).

## 4. Discussion

In this study, metabolome analysis revealed that *sigE* overexpression modified the metabolism from glycogen to PHB during nitrogen starvation in *Synechocystis* 6803 (Supplementary Fig. S5). Genetically engineered *Synechococcus* sp. PCC 7942 containing PHB biosynthetic enzymes of *Alcaligenes eutrophus* accumulates 25% PHB of cellular dry weight under 2-week cultivation in nitrogen-limited conditions with 10 mM acetate.^30^ However, direct use of CO_2_ is desirable from an environmental point of view. In the conditions of our experiments, cells grown photoautotrophically and supplied with 1% (v/v) CO_2_ accumulate up to 1.4% PHB per cellular dry weight (Fig. 5). In a recent study, genetically engineered microalgae *Phaeodactylum tricomutum*, in which the bacterial PHB pathway of *Cupriavidus necator* had been incorporated, accumulated up to 10.6% PHB per algal dry weight after 7 days of nitrogen depletion.^31^ These results, supplementary to ours, suggest that genetically engineered microalga and cyanobacteria are useful for PHB production. The molecular weight and monomer units of PHB are unaltered by *sigE* overexpression (Supplementary Fig. S4). In general, increased PHA synthase activity decreases the molecular weight of PHA.^32^ However, the molecular weight of PHB in *sigE-*overexpressing strain kept similar with that of GT strain, indicating that carbon supply for PHB biosynthesis is enough due to activation of the sugar catabolism by *sigE* overexpression. Type-III PHA synthase, which produces short-chain-length PHAs (including PHB), is present in most of the cyanobacteria,^33^ rendering our NMR results consistent with those of previous studies. Obtaining a range of polyesters, such as other PHAs or polythioesters, is the next goal in cyanobacterial PHA production.

In *Synechocystis* 6803, PHA biosynthetic genes and sugar catabolic genes are co-regulated by SigE, showing that the pathway from glycogen to PHB is regulated at the transcription level (Supplementary Fig. S5). The transcripts of PHA biosynthetic genes peak 6 h after nitrogen starvation, while PHB synthase activity peaks at 48 h after nitrogen depletion.^21^ PHB continuously increases at least 120 h,^21^ indicating translational and post-translational regulation of PHA biosynthetic enzymes. In this way, elucidation of the regulatory mechanism of enzymes in PHA biosynthesis and sugar catabolism is important for PHB production in cyanobacteria. The glycogen levels were similar between the GT and *sigE* overexpression strains after nitrogen starvation (Fig. 2A), indicating that the increase in PHB and the decrease in glycogen are not correlated. We also found that the mRNA of *phaABCE* were less induced by *sigE* overexpression (Fig. 1), however, the proteins of PhaABCE were similarly increased in the *sigE* overexpression strain after nitrogen depletion (Fig. 4). These results suggested that PhaABCE proteins are post-transcriptionally regulated in *Synechocystis* 6803. In terms of four glycogen catabolic genes (*glgX*(slr0237), *glgX* (slr1857), *glgP*(sll1356), and *glgP*(slr1367)), the mRNA and protein levels are also not correlated (Fig. 1 and 2), indicating post-transcriptional regulation of these glycogen catabolic enzymes.

Metabolome analysis suggests that amounts of GDP-mannose and citrate also increased by *sigE* overexpression; thus, reducing the metabolic fluxes of these compounds by genetic modification may further enhance PHB production (Supplementary Fig. S5). In other bacteria, repression of lipopolysaccharide biosynthesis leads to elevated PHB synthesis in *C. necator*.^34^ In addition, multiple regulators are involved in transcriptional control under nitrogen-limited conditions.^35^ *In vitro* studies have shown that Group 1 sigma factor (SigA in *Synechocystis* 6803) possesses stronger transcriptional activity than the Group 2 sigma factors, irrespective of promoter sequences.^36^ Overexpression of *sigE* regulates not only sugar catabolic and PHA biosynthetic genes, but also nitrogen-related genes (Supplementary Fig. S3), suggesting that nitrogen-starvation-induced transcriptional activation depends on the correct balance of transcriptional regulators. Our results also suggest that manipulating multiple transcriptional regulators is important for optimizing PHB production in cyanobacteria.

## Supplementary Data

Supplementary data are available at www.dnaresearch.oxfordjournals.org.

Supplementary Data
