# Supplementary material for: Increased Bioplastic Production with an RNA Polymerase Sigma Factor SigE during Nitrogen Starvation in Synechocystis sp. PCC 6803
Source: DNA Res. 2013 Jul 15;20(6):525–35. doi: 10.1093/dnares/dst028 (PMC3859321; doi:10.1093/dnares/dst028)
Supplement: Supplementary Data [file supp_20_6_525__index.html]

Increased Bioplastic Production with an RNA Polymerase Sigma Factor SigE during Nitrogen Starvation in Synechocystis sp. PCC 6803 — Supplementary Data 

# Increased Bioplastic Production with an RNA Polymerase Sigma Factor SigE during Nitrogen Starvation in *Synechocystis* sp. PCC 6803

## 

Supplementary Data

**Files in this Data Supplement:**

- Supplementary Data - Doc file
- Supplementary Figure 1 - tif file
- Supplementary Figure 2 - tif file
- Supplementary Figure 3 - tif file
- Supplementary Figure 4 - tif file
- Supplementary Figure 5 - tif file
- Supplementary Table 1 - doc file
- Supplementary Table 2 - xlsx file
- Supplementary Table 3 - xlsx file
