# Supplementary material for: Increased Bioplastic Production with an RNA Polymerase Sigma Factor SigE during Nitrogen Starvation in Synechocystis sp. PCC 6803
Source: DNA Res. 2013 Jul 15;20(6):525–35. doi: 10.1093/dnares/dst028 (PMC3859321; doi:10.1093/dnares/dst028)
Supplement: Supplementary Data [file supp_dst028_dst028supp.doc]

**Figure S1.** Metabolic map of glycogen and PHB metabolism in *Synechocystis* 6803. Blue arrow indicates metabolic reaction catalyzed by enzymes whose genes dealt in this study. P designates phosphate.

**Figure S2.** To engineer antibodies against PhaA, B, C, and E, GST-fusion proteins were expressed and purified from *E. coli* BL21 codon plus (Stratagene, La Jolla, CA)*.* GST-PhaB, GST-PhaC, and GST-PhaE were successfully purified, while GST-PhaA was degraded during the purification process. GST-PhaA, -PhaB, -PhaC and -PhaE fusion proteins were resolved by SDS-PAGE on 12% polyacrylamide gels, and stained with Coomassie brilliant blue. Left-pointing arrows (grey) indicate the molecular markers.

**Figure S3.** Quantitative real-time PCR analysis. The plots display mRNA expression levels of *glnB* and *amt1* genes. Data represent means ±SD of results from five independent experiments. The levels were calibrated relative to that of the GT strain under nitrogen-replete conditions (set at 100%). Statistically significant differences between GT and GOX50 are marked by asterisks (Student’s *t-*test; **P*<0.05, ***P*<0.005).

**Figure S4.** NMR Analysis of PHAs. The 270 MHz ^1^H NMR spectra of PHAs from GT (upper panel) and GOX50 (lower panel). Chemical shift of tetramethylsilane was set at 0 ppm. Monomer unit of PHB is designated as 3HB and numbers given in parenthesis indicate the position of carbon within a 3HB monomer.

**Figure S5.** Schematic model of metabolic engineering for PHB production by *sigE* overexpression. Red arrows indicate the pathways enhanced by *sigE* overexpression, deduced from immunoblotting, enzymatic activity, or CE-MS analysis. G1P: glucose-1-phosphate, G6P: glucose-6-phosphate, F6P: fructose-6-phosphate, F1,6P_2_: fructose-1,6-bisphosphate, DHAP: dihydroxyacetone phosphate, GAP: glyceraldehyde-3-phosphate, G1,3P_2_; glycerol-1,3-bisphosphate.
