# Supplementary figures and images for: Increased Bioplastic Production with an RNA Polymerase Sigma Factor SigE during Nitrogen Starvation in Synechocystis sp. PCC 6803
Source: DNA Res. 2013 Jul 15;20(6):525–35. doi: 10.1093/dnares/dst028 (PMC3859321; doi:10.1093/dnares/dst028)

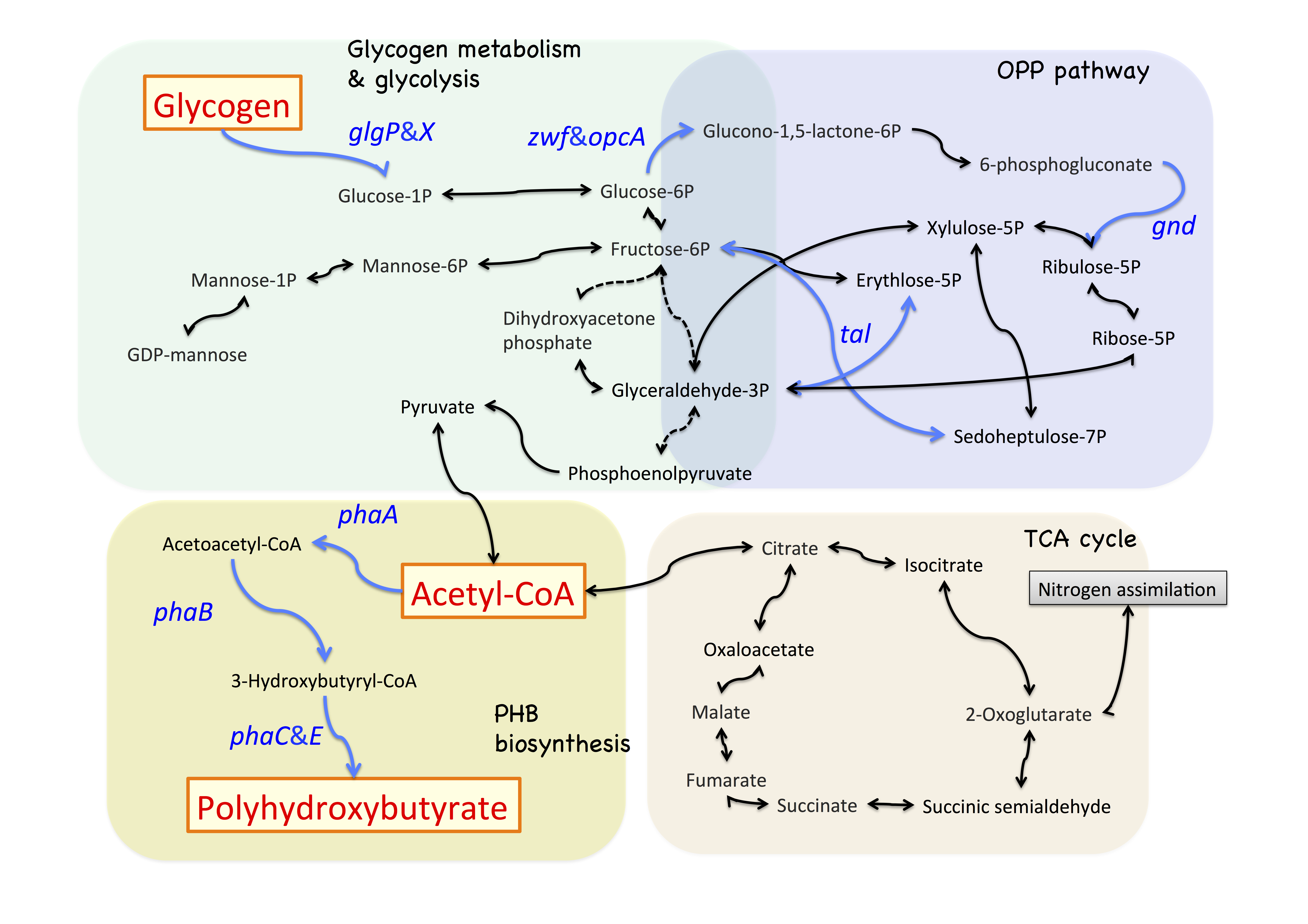

Supplement: Supplementary Data [file supp_dst028_dst028supp_fig1.tif]

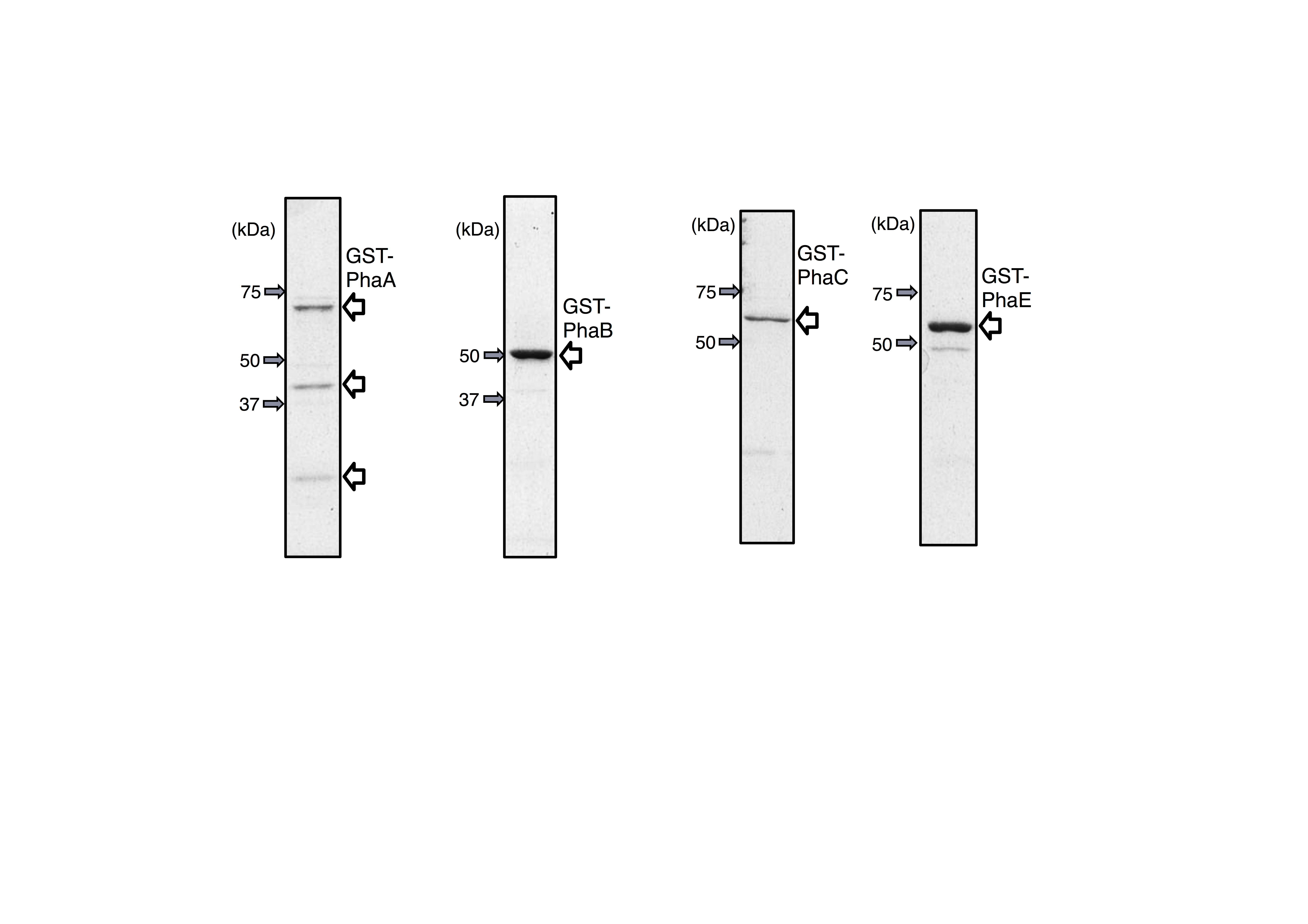

Supplement: Supplementary Data [file supp_dst028_dst028supp_fig2.tif]

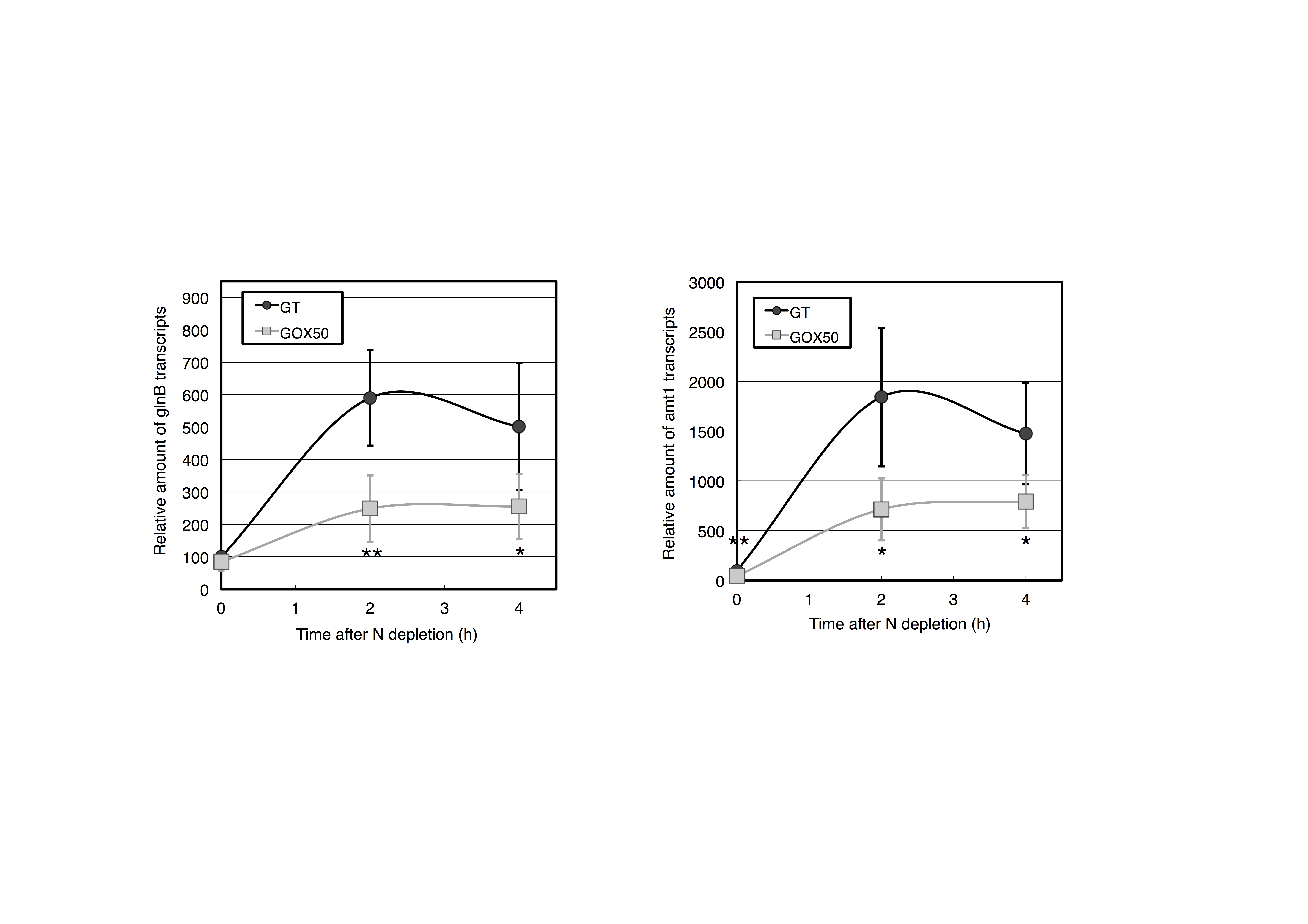

Supplement: Supplementary Data [file supp_dst028_dst028supp_fig3.tif]

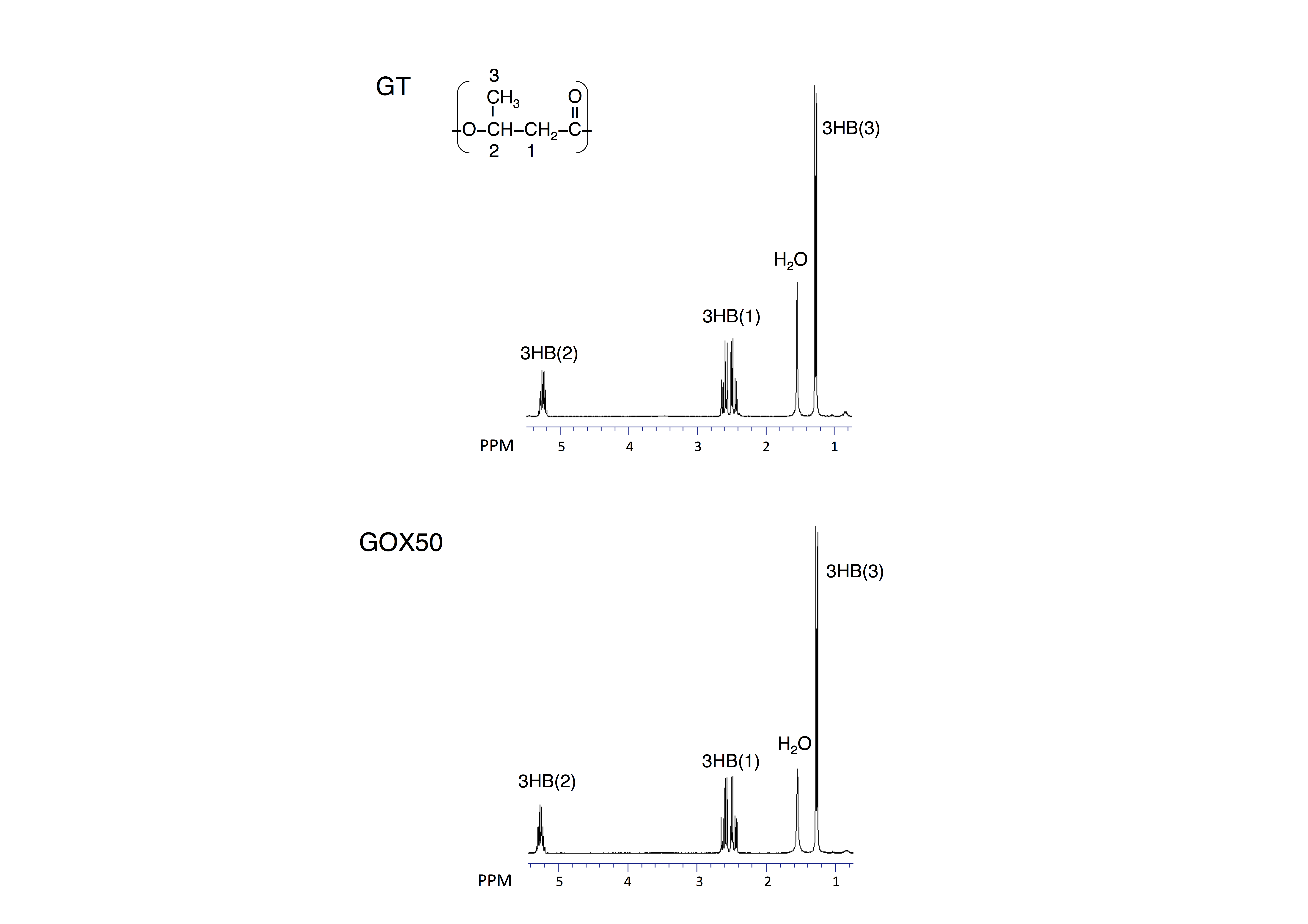

Supplement: Supplementary Data [file supp_dst028_dst028supp_fig4.tif]

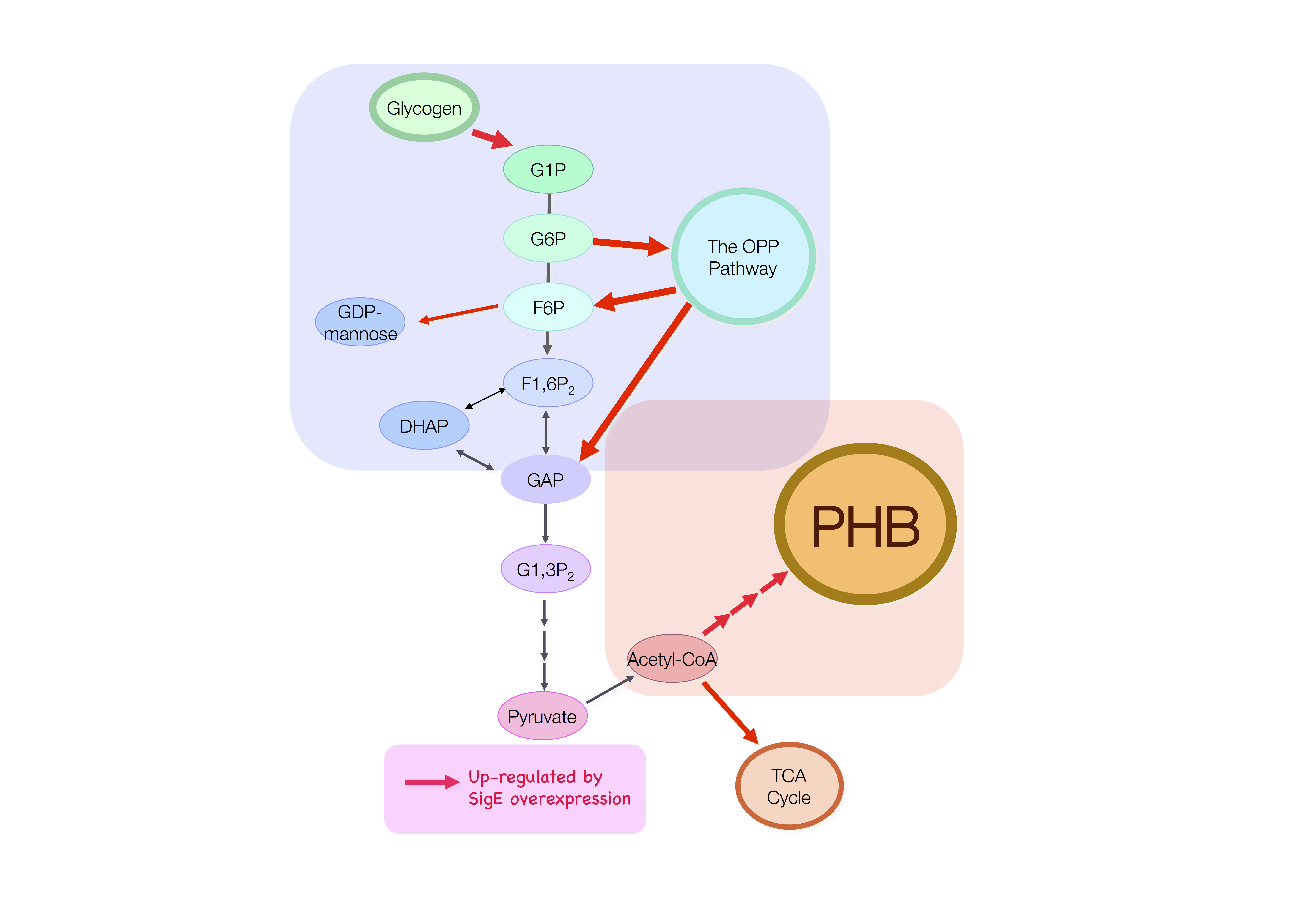

Supplement: Supplementary Data [file supp_dst028_dst028supp_fig5.tif]
