# Supplementary material for: Increased Bioplastic Production with an RNA Polymerase Sigma Factor SigE during Nitrogen Starvation in Synechocystis sp. PCC 6803
Source: DNA Res. 2013 Jul 15;20(6):525–35. doi: 10.1093/dnares/dst028 (PMC3859321; doi:10.1093/dnares/dst028)
Supplement: Supplementary Data [file supp_dst028_dst028supp_table1.doc]

**Table S1. Primers used for purification of GST-tagged proteins and quantitative real-time PCR**

**For GST-tagged proteins**

**Gene**

*phaA* Forward: 5'-ATGAATTCATGGCCGCCCATCCCAAC-3'

Reverse: 5'-GTTGTCGACTCACACCCGCTCTAAGGC-3'

*phaB* Forward: 5'-ATGAATTCATGTTAAGTCTTGGTTTG-3'

Reverse: 5'-GTTGTCGACTTAGGTGTGGTGGGCCCC-3'

*phaC* Forward: 5'-ATGAATTCATGTTTTTACTATTTTTTATCG-3'

Reverse: 5'-GTTGTCGACTCACTGTCGTTCCGATAG-3'

*phaE* Forward: 5'-ATGAATTCATGGAATCGACAAATAAA-3'

Reverse: 5'-GTTGTCGACTTAGCCTGGGTTTGCTTC-3'

**For quantitative real-time PCR**

**Gene**

*rnpB* Forward: 5'-AAAGGGTAAGGGTGCAAAGG-3'

Reverse: 5'-AATTCCTCAAGCGGTTCCAC-3'

*phaA* Forward: 5'-TTTCAGCCGGATAGAATTGG-3'

Reverse: 5'-AGACTTTCCACGGTGGTGTC-3'

*phaB* Forward: 5'-TTTTCCCAAAATTAACCCCC-3'

Reverse: 5'-ACTAATGGCCACAATGGAGC-3'

*phaC* Forward: 5'-GGGCACATTTAGCCTGTGTT-3'

Reverse: 5'-TGGCATCCACCATTAAGTCA-3'

*phaE* Forward: 5'-GGACATGGATGGTTTATGGC-3'

Reverse: 5'-AAGGCGATCGCATAAAACTG-3'

*glgX* Forward: 5'-CTGGTGCGAGATACCAAAGGA-3'

(slr0237) Reverse: 5'-TGGCGGTGGCAATTTCA-3'

*glgX* Forward: 5'-TCCCTCCACTTCGATCAATTTT-3'

(slr1857) Reverse: 5'-CCAAATCCGCCAGGGTAAA-3'

*glgP* Forward: 5'-CGGAACCCTACACCGATGAC-3'

(sll1356) Reverse: 5'-ACCAAACTGCCGGCAATC-3'

*glgP* Forward: 5'-ACCTTTGCCTACACCAATCACA-3'

(slr1367) Reverse: 5'-ATCCACTGACCAGCGCTCTAG-3'

*zwf* Forward: 5'-AGGCATTTCCCTGCGTTTT-3'

Reverse: 5'-GCGGGTACGCAGTTCTGAA-3'

*opcA* Forward: 5'-CAATGCGGCACCGTACTGT-3'

Reverse: 5'-CAGCTTCCATGCGCATACAA-3'

*gnd* Forward: 5'-CCCATCGCCGTGTTTAACC-3'

Reverse: 5'-CGTTCGGCCATGAATTTCTC-3'

*tal* Forward: 5'-CCGGCTGTGACCTGTTGAC-3'

Reverse: 5'-CGGTGTTCCGCAGTTGATC-3'

*glnB* Forward: 5'-GCTGGTTAATGCAGGGATCGT-3'

Reverse: 5'-ACGCTCTGTTTGACCTTTTTGAC-3'

*amt1* Forward: 5'-GCTTTGGCTGGAGCTTTCC-3'

Reverse: 5'-TCGGCCATCAACAAATTTACC-3'
